# Spontaneous regression of a metastatic carcinoma transmitted by a kidney graft

**DOI:** 10.37349/etat.2023.00148

**Published:** 2023-06-30

**Authors:** Mikhail V. Kiselevskiy, Elena G. Gromova, Nikolay A. Kozlov, Svetlana D. Bezhanova, Irina Zh. Shubina

**Affiliations:** Istituto Nazionale Tumori-IRCCS-Fondazione G. Pascale, Italy; ^1^Laboratory of Cell Immunity, N.N. Blokhin National Medical Research Center of Oncology, Ministry of Health of Russia, Moscow 115552, Russia; ^2^Intensive Care Unit, N.N. Blokhin National Medical Research Center of Oncology, Ministry of Health of Russia, Moscow 115552, Russia; ^3^Pathology Department, Division of Morphological and Molecular Genetic Diagnostics of Tumors, N.N. Blokhin National Medical Research Center of Oncology, Ministry of Health of Russia, Moscow 115552, Russia

**Keywords:** Cancer, spontaneous regressions, kidney graft, immunosuppressive therapy

## Abstract

Transmission of a malignancy from a donor’s organ to the recipient of the graft is a rare event, though it is a severe complication that can result in a poor outcome. Usually, immunosuppressive therapy is discontinued and the allograft is removed. However, treatment of patients with the disseminated cancers implies that after the graft removal and cessation of the immunosuppression, radiotherapy, chemotherapy, or immunotherapy with alpha-interferon (INF-α) or interleukin-2 (IL-2) are required. The case report presents a clinical case of a transmitted kidney graft with multiple metastases (MTS) in a 31-year-old woman with the spontaneous regression of the metastatic cancer after transplantectomy and cancellation of the immunosuppressive therapy. Obviously, the determining factor is the recognition of the tumor by the effectors of the antitumor immunity due to the human leukocyte antigen (HLA) mismatch between the donor and the recipient. Therefore, cancellation of the immunosuppressive therapy in cases of transferal of a malignancy with a transplanted organ allows the effectors of the immune system to distinguish the tumor as a foreign tissue and effectively eliminate this neoplasm.

## Introduction

Transmission of a malignancy from a donor’s organ to the recipient of the graft is a rare event occurring in 0.19–0.5% of transplantations [1, 2], though it is a severe complication that can result in a poor outcome [3, 4]. Optimal treatment of the malignant orthotopic solid organ transplant recipients (such as recipients of the grafts of kidney, liver, and pancreas) usually includes discontinuing immunosuppression followed by the removal of the allograft. These manipulations may lead to the regression of the tumor even in cases of the disseminated cancer [5–7]. Gómez-Veiga et al. analyzed 79 cases of *de novo* tumors from the graft and made a conclusion that the developing cancer was less aggressive in comparison to that of the general population and considered a possibility for the cancellation of the transplantectomy [8].

However, treatment of a patient with the disseminated tumor process implies that the graft removal is required, and after cessation of the immunosuppressive therapy, additional treatment is necessary such as radiotherapy, chemotherapy, or immunotherapy with alpha-interferon (INF-α) or interleukin-2 (IL-2) [9–13]. The attempts to use immune checkpoint inhibitors (ICI) in these patients have been also reported, though, despite the clinical effectiveness of this approach, the feasibility of ICI therapy seems doubtful due to a high rate of graft rejection [14].

At the same time, the cases of spontaneous regression of tumors after removal of the graft and cancellation of the immunosuppressive therapy in these patients were described in scientific literature by different authors in the past decades [5–9]. Vincent et al. reported spontaneous regression of metastatic adenocarcinoma transmitted by cadaver kidney graft. The authors defined the phenomenon as “spontaneous immunotherapy” due to the activation of T cells resulting from the cessation of the immunosuppression [15]. We report a case of spontaneous regression of metastatic cancer transmitted by a kidney graft in a 31-year-old woman that was diagnosed 7 months after kidney transplantation.

## Case report

### Patient description

**Figure 1:**
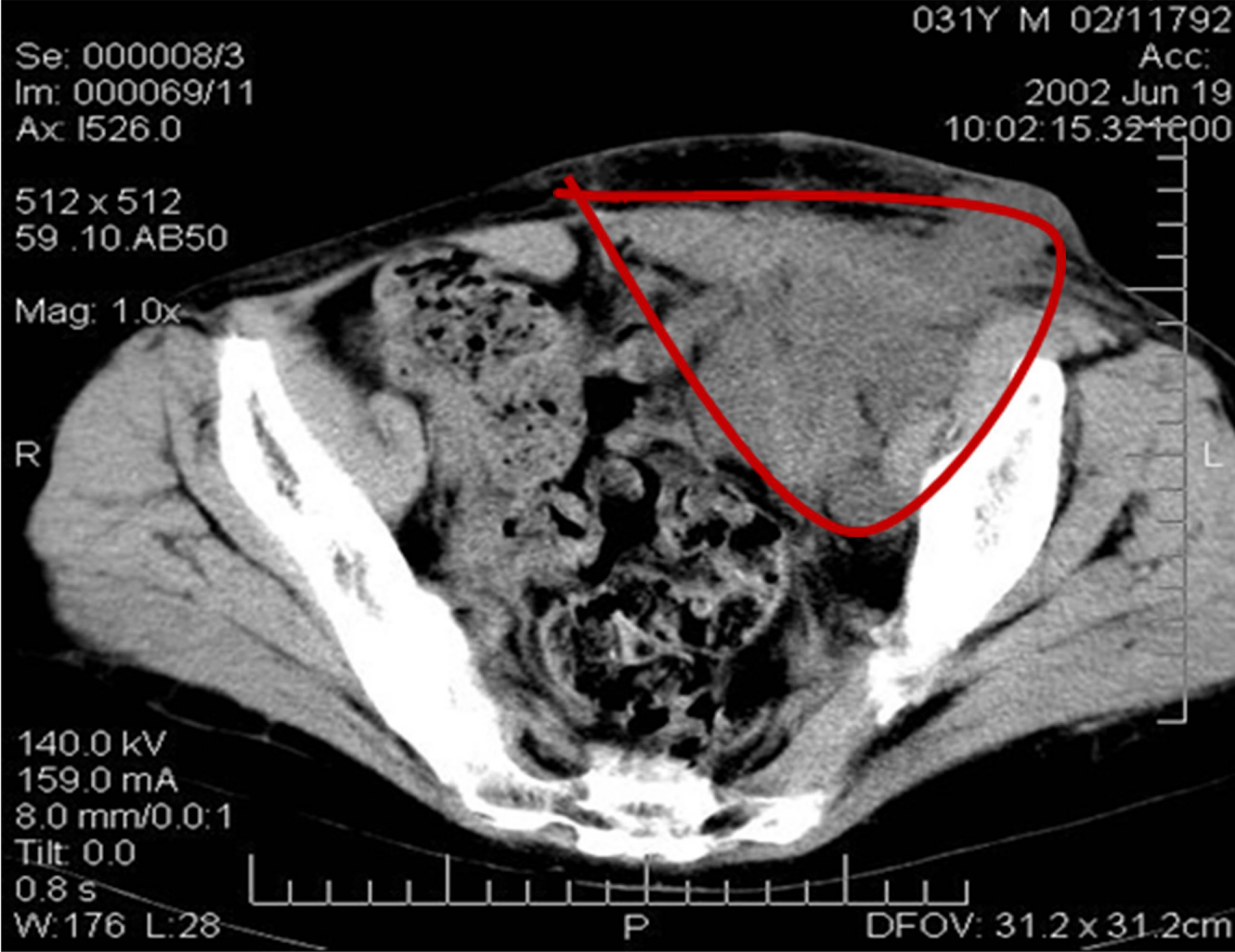
CT image of the patient’s abdomen before the removal of the kidney allograft. Massive tumor conglomerate in the abdominal cavity (the red line delineates the tumor)

Since childhood, the patient (Me., 31 years old) had chronic membranous-proliferative glomerulonephritis which led to secondary renal shrinkage, the development of end-stage chronic renal failure with the need for programmed hemodialysis. On 27 November 2001, heterotopic allotransplantation of a cadaver kidney graft into the left iliac fossa was performed and the patient started immunosuppressive therapy with sandimmun (cyclosporin A). Seven months later the patient complained about the increasing pain in the left iliac area and a gradual increase of the graft size. The examination revealed a tumor in the kidney with metastases (MTS) to the retroperitoneal and inguinal lymph nodes (histopathological examination confirmed high grade renal cell carcinoma). As a result, the immunosuppressants were canceled, and a rejection crisis was initiated by creatinine in the dose of 230 mmol/L. Clinical examination revealed an immobile painful bulky lesion of 15 cm × 20 cm in the right half of the abdomen spreading from the costal arch to the inguinal fold. There was registered a conglomerate of immobile lymph nodes of stone-like density of 8 cm × 6 cm soldering with the inguinal fold; and a fistula with a scanty purulent excretion was detected in the projection of the upper pole of the conglomerate. Ultrasound examination determined a transplant of 11 cm × 10 cm with a thickened heterogeneous parenchyma in the left iliac; thrombosis of the graft vein was possible, as well. Multiple lymph nodes up to 4.5 cm were observed in the left inguinal region above the inguinal fold. A number of hypoechoic areas up to 3 cm were visualized in the soft tissues of the pelvis and pubic area. Computer tomography (CT) scan detected an enlarged transplanted kidney in the pelvic region on the left, and a bulky lesion of 8 cm × 7 cm was found in its parenchyma (Figure 1).

Angiography revealed a vascular-free tumor in the lower third of the graft. No visible changes in the major vessels were found. Ultrasound examination, CT, and radiography did not reveal any mts in the chest. Due to vital indications, the patient underwent palliative transplantectomy and resection of the large omentum.

### Intraoperative picture

**Figure 2:**
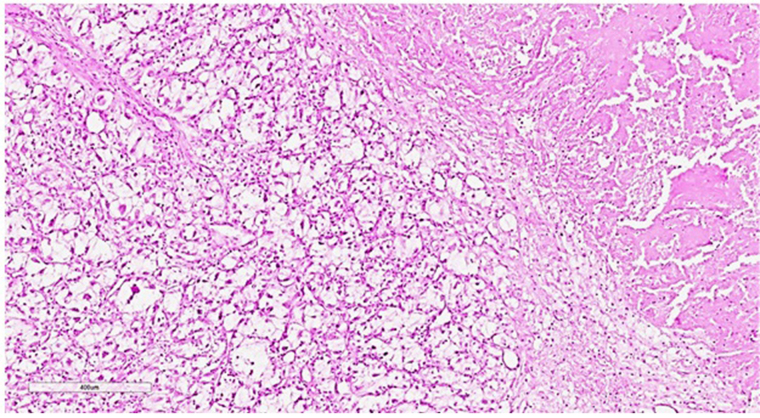
Histopathological slide of the tissue of the transplanted kidney subtotally replaced by the undifferentiated cells with small foci of recognizable clear cell renal cell carcinoma. There is a necrotic area in the upper right part of the image. Hematoxylin-eosin staining, ×60

The size of the transplanted kidney was 14 cm × 11 cm × 6 cm. Normal kidney tissue is determined only in the form of small islands, mainly in the peripheral parts of the organ. Grossly, kidney parenchyma was replaced by grayish-yellow and white tumor tissue with huge foci of hemorrhages and necrosis. Histologically, at the sites with a more preserved tissue, the tumor had the solid (patternless) structure of an undifferentiated epithelioid neoplasm with tiny scattered foci of epithelioid cells with clear cytoplasm and capillary network resembling clear cell renal cell carcinoma. Taking into account these findings, the tumor was conceivably defined as a clear cell renal cell carcinoma Grade 4 [classification of World Health Organization/ International Society of Urological Pathology (WHO/ISUP)] (Figure 2).

Importantly, the tumor tissue was essentially infiltrated mainly by segmented leukocytes, and by lympho-plasmocytic cells in the periphery. There were no significant signs of graft rejection in the surrounding renal tissue. Lymphocytes infiltrated only a few tubules. There were no vast vascular changes. The greater omentum was dense, infiltrated with multiple MTS, the adipose tissue was practically not determined on the cut and the cut surface was represented by a grayish-white homogeneous tissue. The MTS found in the omentum were histologically similar to the graft tumor with clear secondary changes, leukocyte infiltrates, and foci of necrosis. Taking into account the uniformity of reactive changes both in the tumor of the transplanted kidney and in the omentum, these changes were regarded as a manifestation of transplant immunity.

The serous membrane of the transverse colon was covered with disseminated tumor foci up to 3 mm in diameter; ascites reached 500 mL. In the left half of the abdomen, there was registered a tumor-altered transplanted kidney of 10 cm × 12 cm, spreading into the pelvic cavity, that was growing up to the anterior abdominal wall, intimately connected with the sigmoid colon, with the left iliac vessels and left appendages of the uterus, growing into the lumbar muscle on the left. Tumor infiltration was observed along the iliac vessels from the aortic bifurcation to the internal opening of the inguinal canal. Histological examination revealed an undifferentiated malignant tumor with significant lymphoid-plasmacytic and leukocyte infiltration.

### Postoperative examination

Given the “donor’s” origin of the tumor, a decision was made not to perform any specific antitumor treatment, and it was expected that the cessation of immunosuppression might be sufficient to cure the patient. One of the arguments in favor of the “donor’s” genesis of the tumor was the information that the second recipient (M., 28 years old) of the cadaver kidney from the same donor died 4 months after transplantation from a rapidly progressing “undifferentiated tumor” of the transplanted kidney. Regarding histopathological examination of the graft lesion, no any signs of specific tumor type were revealed, as in the described case [16].

The second patient (M., 28 years old), who had been transplanted with the second kidney of the same donor, received the immunosuppressive therapy as the standard treatment after allogenic organ transplantation. After transplantation, an advanced tumor was revealed in the patient’s kidney. Histopathological examination of the tumor biopsy showed the undifferentiated neoplasm, and the immunohistochemical staining for cytokeratins 8, 18, 19 did not identify any expression. The resection of the transplanted kidney was not performed. The rapid progression of the malignant disease led to the patient’s death within the two months. Given the results of this case, the other patient (Me., 31 years old) with the transplanted kidney of the same donor was thoroughly examined and the above-described decisions were made due to the detected graft tumor with the same histopathological characteristics [16].

In the postoperative period, febrile fever and signs of intoxication were observed after cancelling immunosuppressive therapy. However, the focus of infection was not detected and high temperature reaction persisted despite massive therapy with a wide range of antibiotics. Regarding this observation, the clinical picture was considered as “resorption-related fever” caused by the destruction of tumor cells affected by the “transplant immunity”. The patient underwent intermittent hemodialysis within the first four days after the operation. To remove possible tumor decay products, hemodialysis was replaced with hemodiafiltration (27 sessions in total), which led to an improvement of the patient’s performance status and relief of the symptoms.

**Figure 3:**
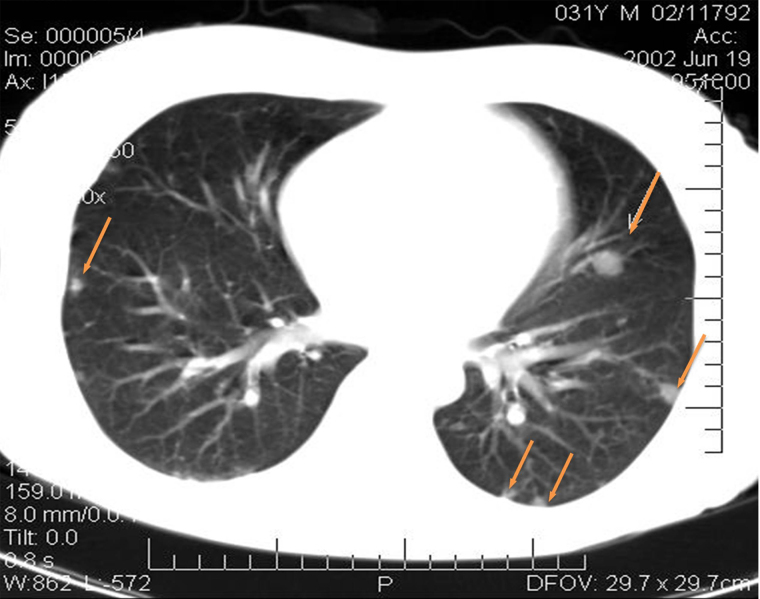
CT images of the patient’s lungs fourteen days after the operation. MTS in the lungs (yellow arrows point at the mts)

Fourteen days after the operation, CT examination demonstrated multiple MTS in both lungs ranging in size from 4 mm to 15 mm (Figure 3); pretracheal lymph nodes with the diameters of 5–8 mm; para-aortic lymph nodes from the LII level, merging into a conglomerate of 36 mm × 39 mm in diameter; iliac lymph nodes merging into a conglomerate of 57 mm × 110 mm; and a lesion located to the right of the rectum 43 mm × 30 mm.

**Figure 4:**
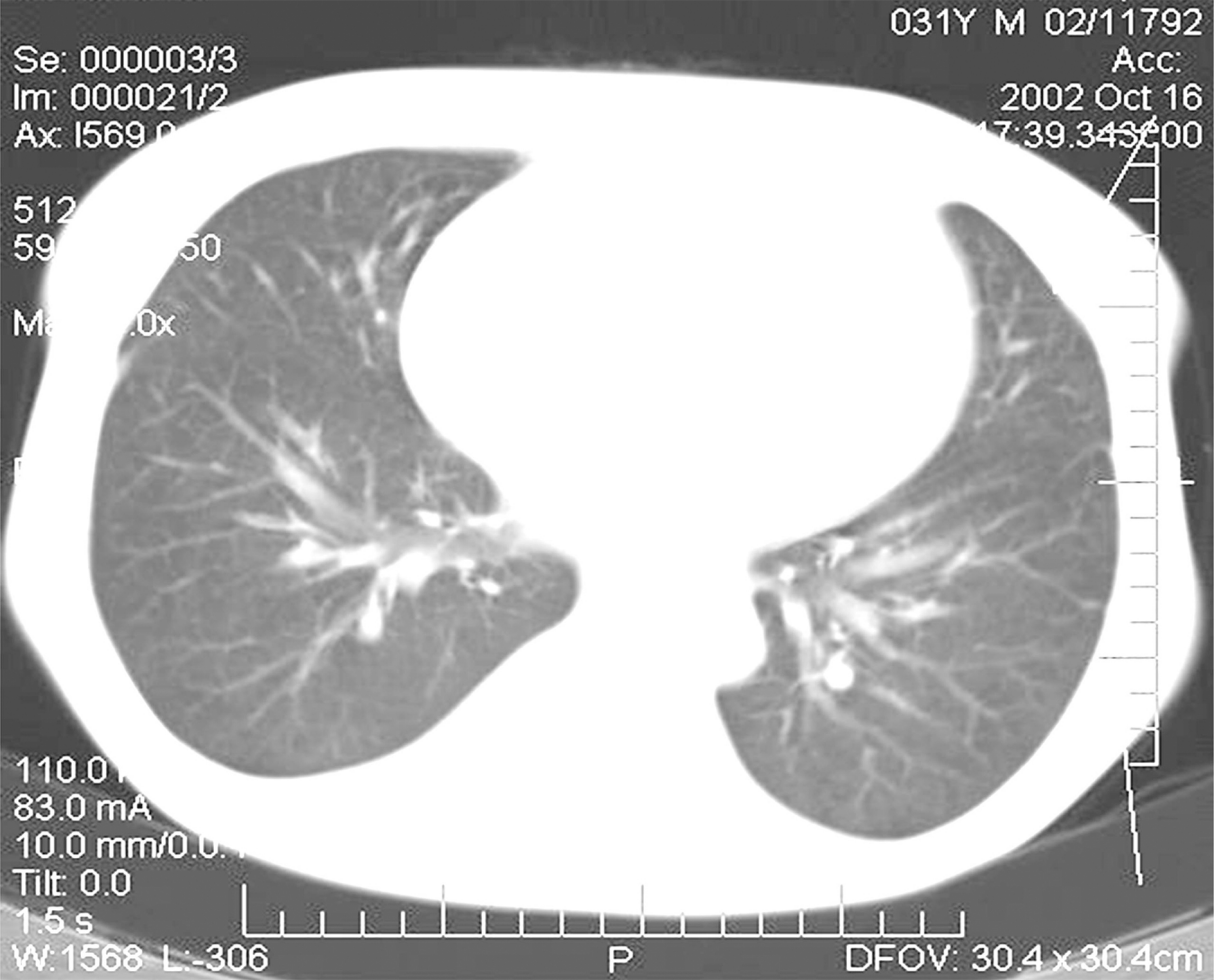
CT images of the patient’s lungs four months after the removal of the kidney allograft: complete regression of several tumor foci and the decrease of the remaining MTS in the lungs

In the postoperative period, there was noted a spontaneous reduction of the tumor conglomerate and infiltrate in the inguinal area on the left to the size of 1 cm × 1 cm. Four months later, the CT scan showed an essential positive trend as compared with that of the previous examination (Figure 4). Practically no multiple lung MTS were detected during this examination. Scars and focal-like formations on average 0.2 cm in diameter developed in the sites of former MTS in the subpleural area in the 11th segment on the right and in the basal segments on the left.

**Figure 5:**
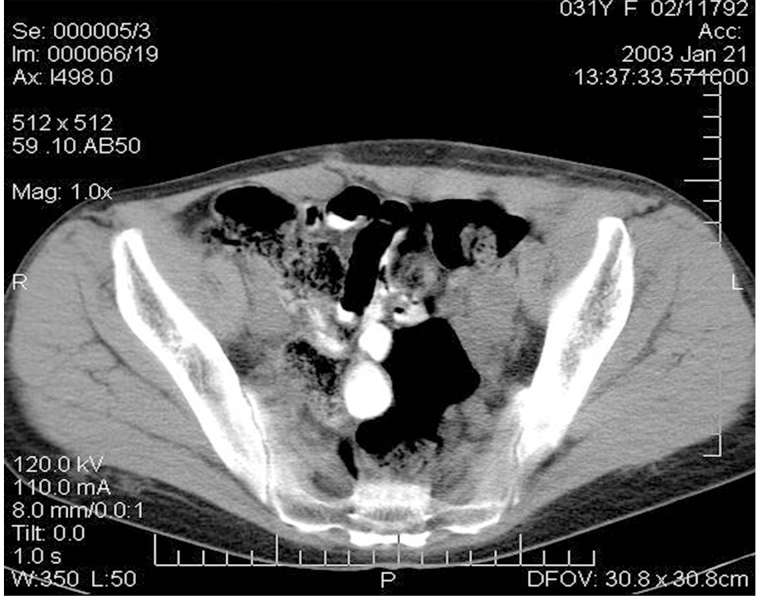
CT image of the patient’s abdomen 6 months after removal of the kidney allograft: complete regression of the lesion in the abdominal cavity

The subcutaneous conglomerate of nodes in the left iliac region was not detected. In the abdominal cavity against the background of the conglomerate of the small intestine there were noted the nodes of 6.2 cm × 3.6 cm in diameter at the left pelvic wall. No other changes were found in the abdominal cavity and pelvic cavity. The patient was discharged from the hospital to be supervised by the urologist and oncologist. The patient’s indications included programmed hemodialysis. Further tumor regression was observed in the follow-up examinations, and complete regression of the tumor foci was registered 6 months after the operation (Figure 5).

Three years later, another kidney transplantation was performed. Over the next 15 years after the second transplantation with the standard immunosuppressive therapy, no symptoms of any malignant neoplasms were detected.

## Discussion

Treatment of patients with advanced cancer that developed during long-term immunosuppressive therapy is a complex and still unresolved problem. The main issue is the use of standard chemo-/radiation therapy or immune-stimulating drugs in these patients after removal of the tumor-affected graft and cessation of immunosuppressive therapy. The regression of tumor foci registered in these cases might be both a consequence of ongoing antitumor therapy and the result of effective immune reactions that could be implemented after discontinued immunosuppression. Previously published data showed that spontaneous regression of the tumor and MTS was observed in approximately half of the initially tumor-affected transplantations after cancellation of the immunosuppressive therapy. The other half of patients received chemo-/radiation therapy since their immune system was considered ineffective in terms of controlling the widespread and extensive MTS [9, 10].

According to some researchers, if spontaneous regression is not observed, the residual neoplasm should be treated with radiation therapy, chemotherapy, or immunotherapy using agents such as INF-α or IL-2. After complete tumor regression, the repeated kidney transplantation should be delayed over the following tumor-free period for at least one year [11–13].

In the case described, the tumor originated from a kidney allograft. Apparently, the standard immunosuppressive therapy promoted the dormant tumor clones to proliferate in the favorable microenvironment, which ultimately resulted in the development of a primary lesion in the allograft and subsequently, the metastatic spread to the greater omentum and lymph nodes and clear manifestation of the malignant disease.

The proof for the assumption that the primary tumor was transmitted by a kidney graft was the fact that another recipient of the same donor’s other kidney developed a tumor in the kidney allograft. In addition, spontaneous regression of tumor MTS after discontinuation of the immunosuppressive therapy and removal of the kidney allograft supported the allogeneic nature of the malignant neoplasm in the above-described patient.

The presented clinical case illustrates the tremendous potential of the immune system, which, despite the immunosuppressive factors of the tumor and long-term immunosuppressive therapy, was able to complete a total regression of the MTS with no signs of the disease recurrence for the following 15 years. Obviously, unlike cases with autologous malignant neoplasms, the immunity of this patient had no barriers to recognize an allogeneic tumor and therefore, even the therapeutically suppressed immunity could realize its potential.

Obviously, the probability of spontaneous tumor regression in patients after transplant removal and cessation of the immunosuppression is determined by various factors, such as the extent of tumor dissemination, the duration of the immunosuppressive therapy, and the initial status of the immunity. However, we believe that the determining factor is the recognition of the tumor by the effectors of the antitumor immunity due to the human leukocyte antigen (HLA) mismatch between the donor and the recipient. Therefore, cancellation of the immunosuppressive therapy in cases of the transmission of a malignancy with the transplanted organ allows the effectors of the immune system to distinguish the tumor as a foreign tissue and effectively eliminate this neoplasm.

## Abbreviations

CT: computer tomography
MTS: metastases

## Declarations

### Copyright

© The Author(s) 2023.
